# Supplementary material for: Effects of Methotrexate and Tofacitinib on Mitochondrial Function and Oxidative Stress in Human Synovial Cells In Vitro
Source: Int J Mol Sci. 2025 Aug 22;26(17):8173. doi: 10.3390/ijms26178173 (PMC12428324; doi:10.3390/ijms26178173)
Supplement: Supplementary file 1 [file ijms-26-08173-s001.zip › ijms-3814029-supplementary.pdf]

SUPPLEMENTARY FILE

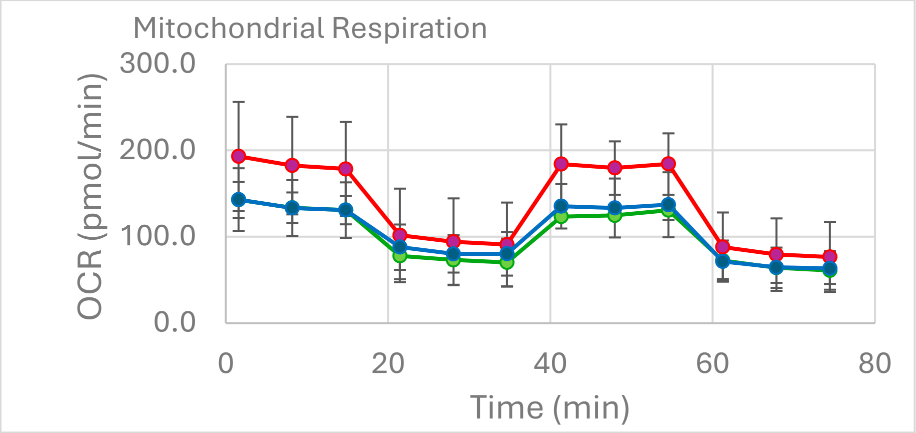

Supplementary Figure S1a. *Representative curves of mitochondrial respiration in SW982 cells*

Legend: Blue line – control SW982 cells (untreated, unstimulated), red line – TNF-alpha stimulated SW982 cells, green line – TNF-alpha stimulated SW982 cells, treated with MTX.

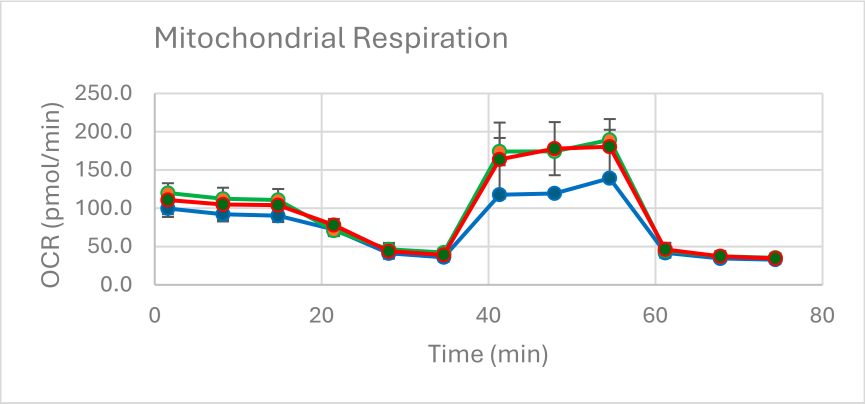

Supplementary Figure S1b. *Representative curves of mitochondrial respiration in SW982 cells*

Legend: Blue line – control SW982 cells (untreated, unstimulated), red line – TNF-alpha stimulated SW982 cells, green line – TNF-alpha stimulated SW982 cells, treated with TFB.

The application of the three inhibitors allowed the calculation of ATP, maximal and reserve respiratory capacity, proton efflux and non-mitochondrial respiration.

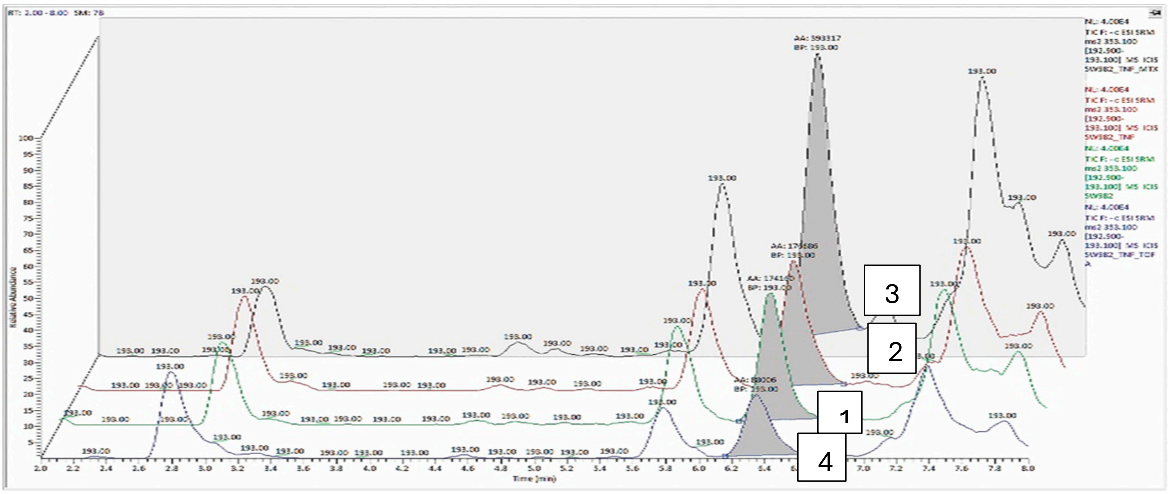

Supplementary Figure S1c. *Chromatographic detection of 8-isoPGF2alpha levels in the supernatant of control SW982 cells (1) TNF-alpha stimulated cell line SW982 (2), TNF – alpha stimulated cell line SW982 and treated with 1µg/ml MTX (3) and TNF-alpha stimulated cell line SW982 and treated with 100 nM TFB (4).*
